# Adult Growth Hormone Deficiency and Metabolic Dysfunction-Associated Steatotic Liver Disease

**DOI:** 10.1007/s13679-026-00692-w

**Published:** 2026-02-19

**Authors:** Stergios A. Polyzos, Alessandro Mantovani, Giovanni Targher

**Affiliations:** 1 First Laboratory of Pharmacology, School of Medicine, Aristotle University of Thessaloniki, Thessaloniki, Greece; 2 Department of Medicine, University of Verona, Verona, Italy; 3 Metabolic Diseases Research Unit, IRCCS Sacro Cuore - Don Calabria Hospital, Negrar di Valpolicella, Italy

**Keywords:** Growth hormone, Metabolic dysfunction-associated steatotic liver disease, Metabolic dysfunction-associated steatohepatitis, Nonalcoholic fatty liver disease, Pituitary, Treatment

## Abstract

**Purpose of Review:**

Growth hormone (GH) deficiency (GHD) in adults presents with metabolic syndrome features, such as abdominal obesity, dysglycemia, atherogenic dyslipidemia and hypertension, which are closely associated with metabolic dysfunction-associated steatotic liver disease (MASLD). This narrative review aims to critically summarize the available data on the link between adult GHD and MASLD, focusing on possible pathophysiological mechanisms, clinical associations and treatment considerations.

**Recent Findings:**

Experimental evidence supports a direct effect of GH on hepatocytes predominantly through the Janus kinase 2 (JAK2)–signal transducer and activator of transcription 5 (STAT5) pathway, which reduces hepatic steatosis. Hepatic steatosis in GHD may also be indirectly affected through the unfavorable effects of GHD on insulin resistance and metabolic syndrome features. Limited data show that low insulin-like growth factor-1 (IGF-1) concentrations may contribute to hepatic fibrosis by acting on hepatic stellate cells. Regarding clinical associations, MASLD is highly prevalent in adults with GHD, and, vice versa, lower or similar circulating GH/IGF-1 concentrations are reported in patients with MASLD compared with those without MASLD. Regarding treatment, favorable effects of recombinant GH (rhGH) on hepatic steatosis and, possibly, on hepatic fibrosis progression in patients with MASLD and GHD or with low IGF-1 concentrations have been demonstrated in interventional studies.

**Summary:**

Existing findings may warrant more specifically designed studies to carefully assess the risk–benefit ratio of rhGH replacement in adults with GHD and MASLD.

## Introduction

Metabolic dysfunction-associated steatotic liver disease (MASLD), formerly known as nonalcoholic fatty liver disease (NAFLD), is a disease of high global prevalence (affecting more than one-third of the general adult population) and limited pharmacological options [1, 2]. MASLD encompasses a spectrum of progressive hepatic phenotypes, ranging from isolated steatosis to metabolic dysfunction-associated steatohepatitis (MASH), formerly known as nonalcoholic steatohepatitis (NASH), cirrhosis and hepatocellular carcinoma (HCC) [3]. The pathogenesis of MASLD is complex and multifactorial [4], with different factors contributing to the disease in each affected individual, underscoring the need for personalized management [5]. The close association of MASLD with features of the metabolic syndrome (MetS), such as obesity, type 2 diabetes mellitus (T2DM), atherogenic dyslipidemia and hypertension is well established and this is also reflected in the last redefinition of the diagnostic criteria of the disease [6]. However, many other factors may contribute to the pathogenesis of MASLD, including, but not limited to, hormones, adipokines, cytokines, endocrine disruptors and infections [7]. In this regard, endocrine and metabolic diseases, beyond obesity and T2DM, seem to manifest MASLD, including polycystic ovary syndrome, hypogonadism, hypothyroidism, Cushing’s syndrome and growth hormone (GH) deficiency (GHD) [8].

GH is secreted in a pulsatile manner by somatotrophic cells of the anterior pituitary gland and exerts its systemic effects primarily by inducing the hepatic production of insulin-like growth factor-1 (IGF-1) [9]. GH is regulated mainly by GH-releasing hormone (GHRH) and somatostatin from the hypothalamus, which stimulate or inhibit, respectively, the secretion of GH [9]. The prevalence of adult GHD is difficult to estimate and is usually underdiagnosed; a reasonable estimate may be approximately 1 per 10,000 population [10]. True adult GHD refers to GH secretion that is lower than the decline in GH secretion due to aging [10]. GHD may occur by multiple causes, such as genetic defects, pituitary compressive tumors (e.g., pituitary adenomas, craniopharyngiomas), hypophysitis, head trauma, radiotherapy or chemotherapy, vascular damage and pharmacotherapy (e.g., immunotherapy) that affect pituitary somatotroph cells producing GH, or may be due to dysfunction of the GH/IGF-1 axis, such as defective hepatic receptors of GH, chronic liver failure or cachexia [11]; 15–20% of cases of adult GHD refers to patients with children-onset GHD being transitioned into adulthood [10]. Since the GH/IGF-1 axis contributes to the carbohydrate and lipid homeostasis, GHD presents with typical MetS features [12–14], which are also common features of MASLD [15]. Experimentally, hepatic steatosis and fibrosis are observed in animal models of GHD and improve after treatment with GH [16]. In line with this, higher rates of MASLD are reported in adults with GHD [17], suggesting that treating GHD is an intriguing option for MASLD in these patients.

Building on this, this narrative review aimed to critically summarize the available data on the link between adult GHD and MASLD, focusing on possible pathophysiological mechanisms, clinical associations and treatment considerations.

## Literature Search

A literature search was conducted in PubMed with no publication date restrictions. Our electronic search strategy was based on a combination of Medical Subject Heading (MeSH) terms and non-MeSH terms, resulting in the following string, which constituted the core of our electronic search: ((“Growth Hormone“[Mesh]) OR (“Growth Hormone-Releasing Hormone“[Mesh]) OR (“Insulin-Like Growth Factor I“[Mesh]) OR (“Insulin-Like Growth Factor II“[Mesh]) OR (“Insulin-Like Growth Factor Binding Proteins“[Mesh]) OR somatotropin OR (“growth hormone deficiency”) OR (“recombinant growth hormone”) OR (“growth hormone analog*”)) AND ((“Non-alcoholic Fatty Liver Disease“[Mesh]) OR (“nonalcoholic fatty liver disease”) OR (“nonalcoholic steatohepatitis”) OR NASH OR NAFLD OR MAFLD OR (“metabolic dysfunction associated fatty liver disease”) OR (“metabolic associated fatty liver disease”) OR (“metabolic dysfunction associated steatotic liver disease”) OR MASLD OR MASH OR (“steatotic liver disease”) OR SLD). Based on this search string, 519 articles were initially retrieved (September 7, 2025); the same search strategy was repeated on December 21, 2025 (before the initial submission) to provide 528 articles. Of them, 50 were selected for providing the best available evidence. Furthermore, 14 more articles were retrieved from the references of the selected articles. During the revision process of the manuscript, we repeated the same search strategy to retrieve 542 published articles (last update: February 8, 2026). As this is a narrative review, some articles outside the search results were included at the authors’ discretion when deemed relevant.

## Pathophysiology

GH is a hormone whose actions are largely opposite to those of insulin and exerts pleiotropic effects on many tissues [9, 18]. A typical example of the antagonistic actions of GH and insulin is the so-called “dawn phenomenon”: early morning peak of GH contributes to an increase in circulating glucose concentrations and a counterbalancing increase in circulating insulin concentrations, targeting to suppress hepatic gluconeogenesis, thus preventing the GH-induced hyperglycemia in healthy individuals, but not in patients with type 1 diabetes mellitus [19].

**Fig. 1:**
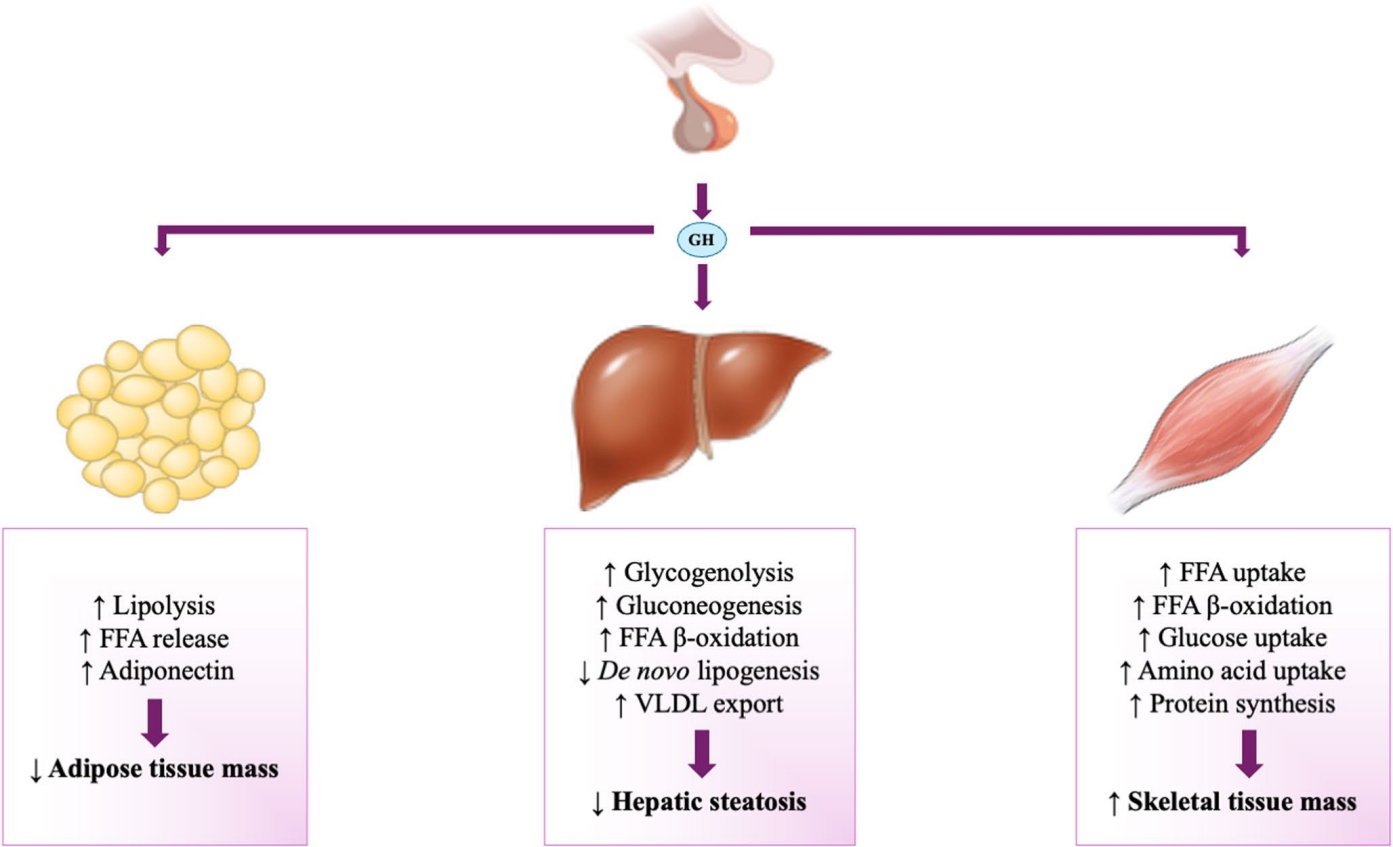
The metabolic effects of GH on the adipose tissue, the liver and the skeletal muscles. In the adipose tissue, GH stimulates lipolysis and the release of FFAs into the circulation, as well as an increase in adiponectin; as a result, adipose tissue mass decreases. In the liver, GH stimulates hepatic glucose production by activating glycogenolysis and gluconeogenesis. GH also increases FFA β-oxidation, decreases *de novo* lipogenesis and increases triglyceride export from the liver by increasing VLDL excretion; as a result, hepatic steatosis is improved. In the skeletal muscle, GH stimulates FFA uptake and β-oxidation, increases glucose and amino acid uptake, thus increasing protein synthesis; as a result, skeletal muscle mass is increased. Abbreviations: FFA, free fatty acids; GH, growth hormone; VLDL, very low-density lipoprotein

In the adipose tissue, GH stimulates lipolysis by activating intracellular hormone-sensitive lipase (HSL), which hydrolyzes triglycerides within adipocytes to release free fatty acids (FFAs) [9] (Fig. 1). GH also contributes to lipolysis through other mechanisms, including modulation of lipid droplet protein expression, such as the cell-death-inducing DFF45-like effector (CIDE-A), and adipokine expression, such as adiponectin [9]. In the liver, GH stimulates hepatic glucose production by activating gluconeogenesis and glycogenolysis [9]. Furthermore, GH increases hepatic FFA β-oxidation, decreases *de novo* lipogenesis and increases the efflux of triglycerides from the liver through increasing the export of very low-density lipoprotein (VLDL) [9, 20]; as a result, triglyceride content decreases in the liver (Fig. 1). In the skeletal muscle, GH stimulates the FFA uptake, but also stimulates FFA β-oxidation [9]. GH also increases glucose and amino acid uptake in skeletal muscles, thereby increasing protein synthesis [21] (Fig. 1). Collectively, GH has largely anabolic effects, except for the adipose tissue, in which it exerts a predominantly catabolic action (lipolysis) [22]. As a result, GH action leads to lower adipose tissue mass and higher skeletal muscle mass.

On the contrary, in adults with GHD, adipose tissue mass is increased (i.e., obesity), while skeletal muscle mass is decreased (i.e., sarcopenia), thereby reducing muscle strength and exercise performance [18, 23, 24]. Fasting hypoglycemia may occur in adults with GHD owing to impaired hepatic glycogenolysis and gluconeogenesis; however, fasting hyperinsulinemia is more common, reflecting systemic IR [23]. Circulating lipid profile is also unfavorable in adults with GHD, typically characterized by high low-density lipoprotein-cholesterol (LDL-C), high triglycerides and low high-density lipoprotein-cholesterol (HDL-C) [18, 23]. All these findings are in line with the observed high rates of MetS and its individual components, which are associated with high cardiovascular morbidity and mortality in adults with GHD [12–14, 18]. It is highlighted that IR is commonly observed in individuals with either GHD or GH excess (acromegaly), occurring due to obesity and dysglycemia in the former and due to the antagonizing effects of GH and insulin in the latter [11, 25]. It should be also emphasized that obesity may initially be a consequence of impaired lipolysis in adult GHD; however, obesity itself may also suppress GH secretion from the pituitary (owing to multiple mechanisms, including increase in insulin, free IGF-1 and FFAs, as well as reduction in adiponectin) and may reduce GH receptors (GHR) in the adipose tissue, leading to a state of GH resistance [9, 11]. Therefore, it seems that there may be a complex pathogenic cycle between obesity and GHD.

### Effects of GHD on Hepatic Steatosis

**Fig. 2:**
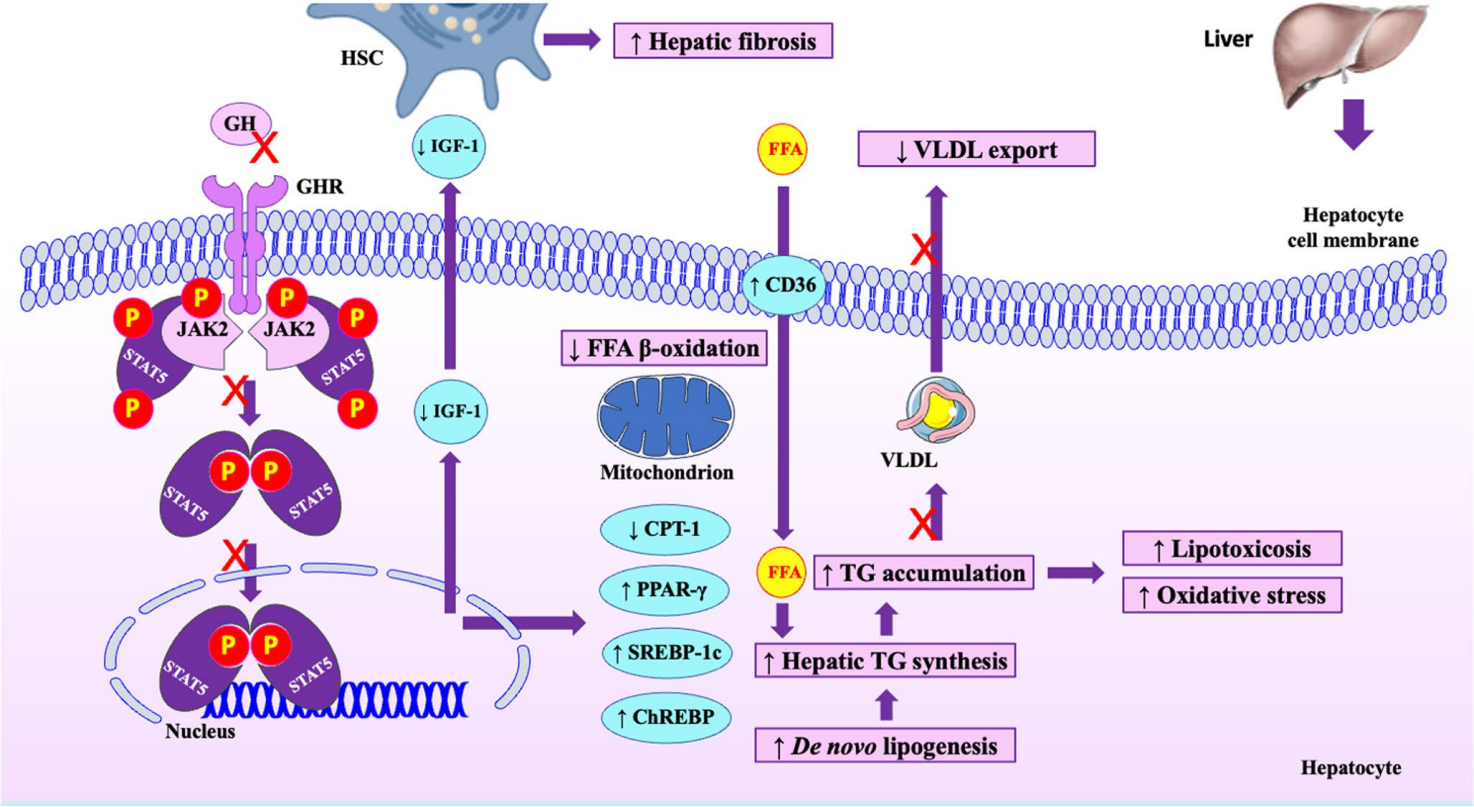
Effects of GHD on the liver. GH exerts a direct effect on hepatocytes via the GHR–JAK2–STAT5 pathway. GHD inactivates the JAK2–STAT5 pathway, leading to upregulation of key lipogenic genes, including SREBP-1c, ChREBP and PPAR-γ, which increase *de novo* lipogenesis. PPAR-γ also upregulates CD36, thus increasing FFA uptake, impairs β-oxidation and decreases VLDL export. CPT-1 is also downregulated, further impairing mitochondrial β-oxidation. These changes lead to intrahepatic TG accumulation, i.e., hepatic steatosis. Impaired β-oxidation may also induce lipid peroxidation, leading to lipotoxicosis and oxidative stress. Furthermore, GHD decreases the production and secretion of IGF-1 by hepatocytes; lower IGF-1 activates HSCs and promotes fibrogenesis, i.e., hepatic fibrosis. Abbreviations: CD36, cluster of differentiation 36; ChREBP, carbohydrate response element binding protein; CPT, carnitine palmitoyltransferase; FFA, free fatty acid; GH, growth hormone; GHD, growth hormone deficiency; GHR, growth hormone receptor; HSC, hepatic stellate cell; IGF, insulin-like growth factor; JAK, Janus kinase; P, phosphate; PPAR, peroxisome proliferator-activated receptor; SREBP, sterol regulatory element-binding protein; STAT, signal transducer and activator of transcription; TG, triglyceride; VLDL, very low-density lipoprotein

Hepatic steatosis is closely associated with MetS and its components, including obesity, dysglycemia, atherogenic dyslipidemia and hypertension, which are commonly observed in adults with GHD [12, 13, 18], as mentioned above. Therefore, GHD may indirectly affect hepatic steatosis. However, GH also directly affects hepatocytes. This hepatic effect is mainly exerted through the GHR–Janus kinase (JAK)2–signal transducer and activator of transcription (STAT)5 pathway [26] (Fig. 2). More specifically, the hepatic-specific deletion of the mouse *Ghr* gene leads to IR, high glucose and FFA concentrations and severe hepatic steatosis, owing to increased *de novo* lipogenesis and increased FFA uptake, as well as reduced hepatic triglyceride export [27–29]. Similar effects were observed in mouse models after deletion of the *Jak2* [30] or *Stat5* [31], which induce GH resistance in the liver. It seems that STAT5 inactivation leads to upregulation of lipogenic genes, including sterol regulatory element-binding protein (SREBP)−1c and carbohydrate response element-binding protein (ChREBP), resulting in hepatic steatosis [29, 32, 33] (Fig. 2). Furthermore, *Jak2* deletion in hepatocytes leads to upregulation of the cluster of differentiation 36 (CD36), an FFA transporter, thereby increasing FFA uptake by hepatocytes [30]. The deletion of *Jak2* also upregulates peroxisome proliferator-activated receptor (PPAR)-γ in hepatocytes, leading to hepatic steatosis via multiple mechanisms, including increased hepatic *de novo* lipogenesis and FFA uptake, as well as impaired β-oxidation [30] (Fig. 2).

Impaired GH signaling also markedly reduces IGF-1 production, which further increases GH secretion, due to the lack of negative feedback of IGF-1 to the pituitary gland [34]; notably, the IGF-1 infusion to *Ghr* knock-out mice did not improve hepatic steatosis, despite normalization of circulating GH concentrations [27]. Similarly, expression of the *Igf1* transgene in *Ggh* knock-out mice did not protect against the development and progression of MASLD, although the restored IGF-1 concentrations improved IR and serum lipid profile, and decreased total body fat [35]. In contrast, treatment with recombinant human GH (rhGH) prevented hepatic steatosis in mice lacking GH [32]. Similarly, a 2-week administration of rhGH to Sprague-Dawley rats previously subjected to hypophysectomy improved hepatic steatosis observed after hypophysectomy [36]. These findings indicate that the above-mentioned effects of GH on hepatocytes are mainly direct and are not mediated by IGF-1. On the other hand, the extrahepatic effects of GHD seem to be primarily mediated by low IGF-1 [35]. Taking all the above into account, the disruption of GH signaling may induce hepatic steatosis. Hepatic steatosis may also be indirectly induced in GHD, through the induction of IR, obesity, dysglycemia and atherogenic dyslipidemia [12, 13, 18].

### Effects of GHD on Hepatic Inflammation and Fibrosis

Experimental data on the potential effects of GH signaling on hepatic inflammation and fibrosis are limited. In one of the above-mentioned studies, hepatic-specific *Jak2* deletion, in addition to hepatic steatosis, led to mild lobular inflammation and fibrosis [30]. Hepatocellular ballooning, a hallmark of hepatic inflammation, was commonly observed in mice lacking GH, but was less common in mice with hepatic-specific deletion of *Stat5* [28]; however, hepatic fibrosis was not evident in this study [28], contrary to the previous one [30]. In another study, hepatic steatosis and fibrosis were shown in a GH-deficient rat model (spontaneous dwarf rats), together with morphological and functional mitochondrial changes [16]. Specifically, hepatic mitochondria were smaller, albeit similar in number to those of control rats. More importantly, the carnitine palmitoyltransferase (CPT)−1, the rate-limiting enzyme for mitochondrial β-oxidation, was decreased and hepatic oxidative stress was increased in GH-deficient rats compared with control rats [16] (Fig. 2). Impaired β-oxidation may result not only in hepatic steatosis, but also in advanced hepatic disease, including MASH, by inducing lipid peroxidation and generating reactive oxidative species [15]. This was more specifically shown in the above-mentioned study with Sprague-Dawley rats, in which rhGH administration promptly reduced hepatic lipid peroxidation and increased glutathione concentrations [36]. Excessive lipid peroxidation after hypophysectomy depletes hepatic nicotinamide adenine dinucleotide phosphate (NADPH), which is used to regenerate glutathione; this results in redox imbalance and oxidative stress in hepatocytes [36]. Regarding hepatic fibrosis, transgenic mice overexpressing IGF-1 mitigated carbon tetrachloride-induced activation of hepatic stellate cells (HSCs), which are key cells contributing to hepatic fibrosis [37]. This effect of IGF-1 resulted in decreased hepatic fibrogenesis and improved hepatocyte regeneration, which were partly mediated by downregulation of transforming growth factor-β and upregulation of hepatocyte growth factor [37]. Notably, other investigators showed that IGF-1 receptors are highly expressed on HSCs and its activation triggered HSC senescence and reduced fibrogenesis in a mouse model of MASH [38] (Fig. 2). The same investigators showed that IGF-1 attenuated hepatic inflammation and fibrosis by improving mitochondrial function and reducing oxidative stress [38]. It seems that IGF-1 affects HSCs both indirectly, via reducing oxidative stress, and directly in a p53-dependent manner, since mice lacking p53, a key intracellular senescence regulator of HSCs, do not benefit from IGF-1 [38].

Notably, some investigators supported that knock-out mice for both *Stat5* and *glucocorticoid receptor* genes were highly susceptible to MASLD-associated HCC: about 60% of these mice developed HCC at 12 months of age [33]. It seems that deleting both genes leads to more advanced MASLD than deleting *Stat5* alone [33]. On the contrary, other investigators reported that *Jak2* knock-out mice fed a high-fat diet did not develop hepatic inflammation or fibrosis (at 4–5 months of age), despite marked hepatic steatosis [39].

Taking all the above into account, more descriptive and mechanistic studies are needed to explore the potential associations between GHD and hepatic inflammation and fibrosis. A plausible hypothesis is that GH acts mainly on hepatocytes, thereby improving hepatic steatosis; consequently, by reducing hepatic steatosis, the progression to MASH and fibrosis could be indirectly attenuated. Furthermore, IGF-1 primarily acts on HSCs, thereby directly decreasing hepatic fibrosis. An important issue could be the potential effect of GH and IGF-1 on Kupffer cells, considered to be key cells in hepatic inflammation. Although experimental data show that GH [40, 41] and IGF-1 [42] favor the switch from M1 (pro-inflammatory) to M2 (anti-inflammatory) phenotype in macrophages, relevant data for Kupffer cells are currently scarce.

## Clinical Associations of Adult GHD and GH with MASLD

For the interpretation of clinical associations between GHD and MASLD, we searched for studies reporting: (a) the rates of MASLD in adults with GHD (Table 1); and (b) circulating GH and/or IGF-1 concentrations in patients with MASLD (Table 2).

**Table 1:** Prevalence of MASLD in observational clinical studies with adult patients with GHD.*

First author, Year, Origin [Reference]†	Study design	Study groups (*n*)	BMI (kg/m^2^)§	Method(s) of diagnosis of NAFLD/MAFLD/MASLD	Rate of patients with NAFLD/MAFLD/MASLD (%)	Main findings
Das 2022 India [43]	Cohort	Patients with Sheehan’s syndrome (29) vs. controls without Sheehan’s syndrome (26)	23.3 ± 5.0 vs. 24.3 ± 4.2	TE (CAP and LS)	63 vs. 65 (total) 51 vs. 30 (severe steatosis)	Higher rates of severe hepatic steatosis in patients than controls. GHD was independently associated with hepatic steatosis.
Diniz 2014 Brazil [44]	Case-control	Patients with genetic isolated GHD (22) vs. controls (22)	24.0 ± 5.0 vs. 25.3 ± 4.2	US (hepatic steatosis)	61 vs. 29^a^	Higher rates of hepatic steatosis in patients with than without GHD.
Gardner 2012 UK [45]	Case-control	Patients with GHD (28) vs. controls (24)	27.8 (24.7–34.7) vs. 27.9 (25.1–32.1)^#^	MRI (intrahepatic lipids)	32 vs. 50	Not significantly different rates of hepatic steatosis between patients and controls.
Hong 2011 South Korea [46]	Case-control	Patients with GHD (34) vs. controls (40)	25.2 ± 0.5 vs. 22.1 ± 0.2^a^	US (hepatic steatosis)	71 vs. 13^a^	Higher rates of hepatic steatosis in patients with than without GHD.
Hwang 2023 South Korea [47]	Cross-sectional	Patients with NF pituitary adenomas (278): GHD (145) vs. non-GHD (133)	24.8 ± 3.3 vs. 23.4 ± 3.3^a^	HSI (hepatic steatosis)	37 vs. 21^a^	Higher rates of hepatic steatosis in patients with than without GHD.
Ichikawa 2003 Japan [48]	Cross-sectional	Patients with hypopituitarism with GHD (13) vs. without GHD (5)	23.5 ± 2.2 vs. 21.3 ± 1.2	CT (hepatic steatosis)	54 vs. 0^a^	Higher rates of hepatic steatosis in patients with than without GHD.
Kang 2021 South Korea [49]	Case-control	Patients with childhood-onset hypopituitarism (76) vs. controls (74)	24.7 ± 4.7 vs. 23.9 ± 1.0	MRI (hepatic fat fraction) and TE (CAP and LS)	71 vs. 31^a^	Higher rates of hepatic steatosis in patients than controls. The rate of presumed hepatic fibrosis in patients was 34% (NA in controls).
Meienberg 2016, UK [50]	Case-control	Patients with GHD (22) vs. controls (44)	28.5 ± 5.0 vs. 27.9 ± 4.3	MRS (intrahepatic lipids)	23 vs. 16	Not significantly different rates of hepatic steatosis between patients and controls.
Nishizawa 2012 Japan [51]	Case-control	Patients with GHD (66) vs. controls (83)	25.0 ± 4.9 vs. 25.2 ± 3.5	US (hepatic steatosis) Liver biopsy (selected patients only)	77 vs. 12^a^	Higher rates of hepatic steatosis in patients than controls.

*: Original nomenclature of the liver disease (i.e., NAFLD, MAFLD, MASLD) was kept in the table for each study, for the sake of consistency

†: Studies are sorted in alphabetical order, primarily according to the first author surname and secondarily according to the publication year

a: Statistically significant between groups

*§: Data are presented as mean ± standard deviation, except if it is differently indicated*

*#: Median (range)*

*Abbreviations*: *BMI* body mass index, *CAP* controlled attenuation parameter, *CT* computerized tomography, *GHD* growth hormone deficiency, *HSI* hepatic steatosis index, *LS* liver stiffness, *MAFLD* metabolic dysfunction-associated fatty liver disease, *MASLD* metabolic dysfunction-associated steatotic liver disease, *MRI* magnetic resonance imaging, *MRS* magnetic resonance spectroscopy, *n* number, *NA* not available, *NAFLD* nonalcoholic fatty liver disease, *NF *non-functioning*, TE* transient elastography, *US* ultrasonography

**Table 2:** Main characteristics and outcomes of observational clinical studies on the associations between circulating GH and/or IGF-1 concentrations and MASLD.*

First author, Year, Origin [Reference]†	Study design	Study groups (*n*)	Method(s) of diagnosis of NAFLD/MAFLD/MASLD	BMI (kg/m^2^)§	Circulating GH: basal, nadir or peak (ng/ml); AUC (ng/ml*min) ¥§	Circulating IGF-1 (units are indicated per study)§	Main findings
Arturi 2011 Italy [52]	Cross-sectional (data from CATAMERI study)	Individuals with ≥ 1 cardiometabolic factor (503): NAFLD (308) vs. non-NAFLD (195)	US (hepatic steatosis)	31.6 ± 5.8 vs. 27.7 ± 4.4^a^	NA	139 ± 57 vs. 168 ± 73 (ng/ml)^a^	IGF-1 was lower in patients with NAFLD than non-NAFLD group.
Chisima 2017 Japan [53]	Cross-sectional	Patients with NAFLD (222) and with chronic HCV (55)	Liver biopsy	26.9 (19.7–43.3) vs. 22.7 (15.7–29.6)^ß, a^	NA	NA	GH was lower and IGF-BP3 was higher in patients with severe steatosis than those with mild-to-moderate steatosis (within NAFLD patients, but not within HCV patients). IGF-1 and IGF-BP3 were lower in patients with F4 than those with F1-F3 (within NAFLD patients, but not within HCV patients). GH, IGF-1 and IGF-BP3 were not associated with hepatocellular ballooning.
Colak 2012 Turkey [54]	Case-control	Patients with NAFLD (92) vs. non-NAFLD (51)	US (hepatic steatosis) Liver biopsy (patients only)	31.8 ± 5.3 vs. 24.6 ± 3.7^a^	NA	133.3 ± 76.7 vs. 126.8 ± 71.6 (ng/ml)	IGF-1 was similar between groups. IGF-BP5 was higher in patients with NAFLD than non-NAFLD group. IGF-BP5, but not IGF-1, was higher in patients with definite NASH than those with SS or possible NASH. IGF-1 was lower, whereas IGF-BP5 higher in NAFLD patients with F2-3 than those with F0-1.
Dichtel 2017 USA [55]	Cross-sectional	Patients with liver biopsies (142): NASH (80) vs. SS (41) vs. non-NAFLD (21)	Liver biopsy	41.4 ± 9.9 vs. 43.9 ± 6.8 vs. 42.7 ± 8.3	NA	109 ± 45 vs. 136 ± 57 (ng/ml)^a^ (NASH vs. SS/non-NAFLD)	IGF-1 was lower in patients with NASH than SS/non-NAFLD. IGF-1 was lower in patients with than without lobular inflammation or hepatocellular ballooning. IGF-1 was lower in NAFLD patients with F2-4 than those with F0-1. IGF-1 was similar between patients with and without hepatic steatosis.
Fellinger 2023 Austria [56]	Cross-sectional	Apparently healthy individuals (59): NAFLD (16) vs. non-NAFLD (43)	MRS (intrahepatic lipids)	27.8 ± 2.4 vs. 23.2 ± 3.1^a^	GH nadir: 0.015 (0.015–0.090) vs. 0.014 (0.058–0.268)^ß, a^ GH AUC: 16.8 (7.2–32.0) vs. 47.7 (21.6–143.0)^ß, a^	62.0 ± 16.8 vs. 70.3 ± 17.3 (% ULN)	GH nadir and AUC (after OGTT) were lower in those with than without NAFLD. IGF-1 was similar between groups.
Fusco 2012 Italy [57]	Cross-sectional	Patients with obesity (115): NAFLD (65) vs. non-NAFLD (50)	US (hepatic steatosis)	33.7 ± 2.5 vs. 32.0 ± 1.7	GH peak: 9.0 ± 5.8 vs. 11.5 ± 7.1^a^	17.1 ± 7.3 vs. 20.5 ± 6.9 (mM)^a^	IGF-1 and GH peak (after GHRH test) were lower in patients with NAFLD than non-NAFLD group. GHBP and IGF-BP3 were higher in patients with NAFLD than non-NAFLD group.
García-Galiano 2007 Spain [58]	Case-control	Patients with severe obesity (36) vs. non-obese controls (12)	US (hepatic steatosis) Liver biopsy (patients only)	51.0 ± 7.0 vs. 24.0 ± 3.5^a^	NA	143 ± 11 vs. 200 ± 8 (ng/ml)^a^	IGF-1 was lower in patients with severe obesity than non-obese controls. IGF-1 was lower in patients with severe than mild steatosis. IGF-1 was lower in patients with definite NASH than those with possible NASH or non-NASH.
Huang 2022 China [59]	Cross-sectional	Patients with PSIS (93): NAFLD (47) vs. non-NAFLD (46)	US (hepatic steatosis)	25.4 ± 5.2 vs. 22.0 ± 3.7^a^	NA	6.4 (3.9–11.9) vs. 11.6 (7.1–19.6) (nmol/l)^ß, a^	IGF-1 was lower in patients with NAFLD than non-NAFLD group.
Ichikawa 2007 Japan [60]	Cross-sectional	Patients with NAFLD (52): steatosis grade 2–3 vs. steatosis grade 0–1	Liver biopsy	28.7 ± 3.6 vs. 29.2 ± 4.4	GH basal: 0.33 ± 0.36 vs. 0.30 ± 0.37	129 ± 45 vs. 192 ± 128 (ng/ml)^a^	IGF-1, but not basal GH or IGF-BP3, was lower in patients with steatosis grade 2–3 than 0–1. IGF-1 was lower in patients with than without portal fibrosis, but similar in those with and without pericellular or bridging fibrosis. IGF-1 was similar in patients with and without hepatocellular ballooning.
Koehler 2012 USA [61]	Cross-sectional	Patients with obesity (160): NASH/F ≥ 2 (12) vs. NASH/F < 2 (60) vs. SS (72) vs. non-NAFLD (16)	Liver biopsy	50.4 ± 8.7 vs. 47.1 ± 9.1 vs. 45.8 ± 7.3 vs. 47.3 ± 7.0	GH basal: 0.14 (0.04–0.22) vs. 0.10 (0.03–0.37) vs. 0.21 (0.09–0.49) vs. 0.45 (0.16–1.16)^ß, a^	Free IGF-1: 0.93 (0.73–1.08) vs. 0.99 (0.72–1.17) vs. 0.93 (0.83–1.13) vs. 0.98 (0.84–1.06) (ng/ml)	Basal GH was lower in NASH than non-NASH groups. Free IGF-1 was similar between groups.
Lonardo 2002, Italy [62]	Case-control	Patients with NAFLD (61) vs. non-NAFLD (104)	US (hepatic steatosis)	28.8 ± 0.6 vs. 24.6 ± 0.3^a^	GH basal: 0.03 ± 0.09 vs. 0.10 ± 0.21^a^	NA	Basal GH was lower in NAFLD than non-NAFLD group.
Matsumoto 2018 Japan [63]	Cross-sectional	Individuals undergoing check-up (338): NAFLD (89) vs. non-NAFLD (249)	US (hepatic steatosis)	26.1 ± 3.0 vs. 22.4 ± 2.8^a^	NA	169 (57) vs. 161 (53) (ng/ml)^c^	IGF-1 was similar between groups.
Osganian 2022 USA [64]	Case-control	NASH/F ≥ 1 (82) vs. NASH/F0 (72) vs. SS (88) vs. non-NAFLD (76)	Liver biopsy	47.9 ± 7.3 vs. 47.4 ± 7.6 vs. 46.1 ± 7.2 vs. 45.7 ± 6.6	NA	NA	Hepatic expression of IGF-1, but not of GHR or IGF-1 receptor, was lower in patients with NASH than those with SS.
Polyzos 2020 Greece [65]	Case-control	Study #1: NAFLD (18) vs. non-NAFLD (14) Study #2: NASH (16) vs. SS (15) vs. non-NAFLD (with obesity; 26) vs. non-NAFLD (lean; 24)	Study #1: US (hepatic steatosis) Study #2: US (hepatic steatosis) Liver biopsy (patients only)	Study #1: 29.9 (27.5–31.4) vs. 24.1 (22.8–26.2)^ß, a^ Study #2: 34.8 (29.7–39.8) vs. 30.3 (29.4–36.3) vs. 30.4 (28.9–32.2) vs. 25.5 (23.9–26.8)^ß, a^	NA	Study #1: 173 ± 55 vs. 184 ± 79 (ng/ml) Study #2: 207 ± 106 vs. 209 ± 64 vs. 260 ± 81 vs. 182 ± 48 (ng/ml)^a^	Study #1: IGF-1 was similar between groups. Total and intact IGF-BP3 and IGF-BP4 were similar between groups. Study #2: IGF-1 was similar in patients with NASH and SS. IGF-1 was higher in control groups with obesity than lean control group. Total IGF-BP3 and total IGF-BP4 were similar between groups. Intact IGF-BP3 and intact IGF-BP4 were higher in NASH.
Rufinatscha 2018 Austria [66]	Cross-sectional	Patients with obesity (29): NASH (15) vs. SS (14)	Liver biopsy	45.4 ± 5.4 vs. 47.6 ± 10.6	NA	NA	Hepatic expression of IGF-1, but not of GHR, was lower in patients with NASH than those with SS.
Savastano 2011 Italy [67]	Cross-sectional	Patients with obesity/overweight (48): NAFLD (27) vs. non-NAFLD (21)	US (hepatic steatosis)	37.4 ± 5.8 vs. 32.7 ± 5.8	NA	138 ± 54 vs. 204 ± 95 (ng/ml)^a^	IGF-1 and IGF-BP1 were lower in patients with NAFLD than non-NAFLD group. IGF-BP3 was similar between groups.
Sesti 2013 Italy [68]	Cross-sectional (data from CATAMERI study)	Individuals with ≥ 1 cardiometabolic factor (473): NAFLD (234) vs. non-NAFLD (239)	US (hepatic steatosis)	32.3 ± 5.7 vs. 27.9 ± 5.1	NA	147 ± 57 vs. 172 ± 70 (ng/ml)^a^	IGF-1 was lower in patients with NAFLD than non-NAFLD group.
Sumida 2015 Japan [69]	Case-control	Patients with NAFLD (199) vs. non-NAFLD (2911)	Liver biopsy (patients only)	29.8 (24.7–30.0)^ß^ vs. NA	NA	112 (87–150) vs. 121 (107–141) (ng/ml)^a^	IGF-1 was lower in patients with NAFLD than non-NAFLD group. IGF-1 was lower in patients with NASH than those with SS. IGF-1 was lower in NAFLD patients with F3-4 than those with F0-2. IGF-1 was similar between patients with SS and non-NAFLD group.
Xu 2012 China [14]	Cross-sectional	Patients with NAFLD (1667) vs. non-NAFLD (5479)	US (hepatic steatosis)	24.6 ± 3.0 vs. 22.8 ± 3.0^a^	GH basal: 0.02 (0.01–6.01) vs. 0.11 (0.02–7.09)^a^	NA	Basal GH was lower in NAFLD than non-NAFLD group.

*: Original nomenclature of the liver disease (i.e., NAFLD, MAFLD, MASLD) was kept in the table for each study, for the sake of consistency

†: Studies are sorted in alphabetical order, primarily according to the first author surname and secondarily according to the publication year

a: Statistically significant difference between groups

¥: GH nadir, GH peak and GH AUC were measured during a stimulation test for GH

§: Data are presented as mean ± standard deviation, except if it is differently indicated

ß: Median (25th – 75th percentile)

c: Median (interquartile range)

Abbreviations: *AUC* area under the curve, *BMI* body mass index, *CATAMERI* Catanzaro Metabolic Risk Factors, *F* fibrosis stage, *GH* growth hormone, *GHBP* growth hormone binding protein, *GHRH* growth hormone releasing hormone, *GHR* growth hormone receptor, *HCV* hepatitis C virus, *IGF-1* insulin-like growth factor-1, *IGF-BP* insulin-like growth factor-binding protein, *MAFLD* metabolic dysfunction-associated fatty liver disease, *MASLD* metabolic dysfunction-associated steatotic liver disease, *MRS* magnetic resonance spectroscopy, *n* number, *NA* not available, *NAFLD* nonalcoholic fatty liver disease, *NASH* nonalcoholic steatohepatitis, *OGTT* oral glucose tolerance test, *PSIS* pituitary stalk interruption syndrome, *RCT* randomized controlled trial, *SS* simple steatosis, *ULN* upper limit of normal, *US* ultrasonography

### Rates of MASLD in Adults with GHD

Relevant studies reporting the comparative rates of MASLD in adults with and without GHD are summarized in Table 1 [43–51]. All retrieved studies were observational (cross-sectional or case-control) from Asia, Europe and South America. The first of them was published in 2003 [48]. In most published studies, the rate of MASLD was higher (almost or more than doubled) in adults with GHD than in those without GHD (Table 1). The diagnosis of MASLD for all retrieved studies was based on imaging methods or non-invasive tests (NITs). In contrast, liver biopsy was used only in a subset of selected patients with MASLD in one study [51]. Notably, body mass index (BMI), a major confounder in MASLD studies, was similar between groups in most, but not all, studies (Table 1). Another limitation is that, in some studies (e.g. [49–51]),, GHD was not isolated (Table 1), so their results may be affected by the deficiency of other pituitary hormones (e.g., secondary hypothyroidism and/or hypogonadism). However, in a cohort study of patients with Sheehan’s syndrome, of whom 86% had GHD, BMI and GHD were the main independent associates of MASLD after 9.8 ± 6.8 years of follow-up; in this study, MASLD was present in 63% of patients, half of whom had presumed severe hepatic steatosis [43]. This may imply that even in cases without isolated GHD, GHD plays a pivotal role in the development of MASLD.

In line with the above, in a meta-analysis of 10 observational studies, the prevalence of MASLD in patients with GHD was 51% (95% CI 39–63%) [17], which is higher than the prevalence of MASLD in the general adult population (30.1%; 95% CI 27.9–32.3%) [70]. In the same meta-analysis, the prevalence of MASH was 18% (95% CI 5–31%), which is also higher than that in the general population (5.3%) [70].

It should be emphasized that GHD is also associated with MetS components, which may partly account for the high cardiovascular mortality in patients with GHD [18, 71]. As mentioned above, MetS and its individual components are also associated with MASLD [3, 6].

Important data on the association between defects in the GH pathway and MASLD may also be obtained from studies of patients with Laron syndrome, a rare genetic disorder inherited in an autosomal recessive manner and caused by inactivating mutations in the *GHR* gene [72]. Laron syndrome is regarded as the prototype of GH resistance; circulating IGF-1 concentrations are very low or undetectable, whereas GH concentrations are commonly elevated, owing to the lack of IGF-1 inhibition at the pituitary gland [72]. Adult patients with Laron syndrome are characterized by very low stature (dwarfism), mainly due to IGF-1 deficiency; they also exhibit IR and high rates of obesity, T2DM, atherogenic dyslipidemia and arterial hypertension [73]. More than half of untreated patients with Laron syndrome have hepatic steatosis, assessed by ultrasonography (US) [73, 74]. Although there are no specifically designed trials on the effect of IGF-1 replacement on hepatic steatosis in patients with Laron syndrome, the rate of hepatic steatosis in those under IGF-1 replacement is about 40%, which may imply a small to null effect of IGF-1 replacement on hepatic steatosis [74]. This is expected, as IGF-1 replacement does not restore the GHR–JAK2–STAT5 signaling in hepatocytes (section #3). Notably, all patients with Laron syndrome also have obesity, which does not respond to IGF-1 replacement [73].

Overall, MASLD is a highly prevalent condition in adults with GHD. Even in the setting of hypopituitarism, GHD seems to play a key role in the development of MASLD. These associations could be attributed to shared MetS components observed in both GHD and MASLD, as well as to a direct effect of GHD on MASLD.

### Circulating GH and/or IGF-1 Concentrations in Adults with MASLD

Relevant studies with largely unselected MASLD populations, in which circulating GH and/or IGF-1 concentrations were compared between patients with MASLD and controls or between patients with different severity of MASLD, are summarized in Table 2 [14, 52–69]. All retrieved studies were observational (cross-sectional, case-control or cohort) and were from Europe, North America and Asia. The first relevant study was published in 2002 [62]. In most studies, IGF-1 was lower in patients with MASLD than in those without MASLD; however, some studies reported similar IGF-1 concentrations between patients and controls [54, 56, 63, 65] (Table 2). Data regarding IGF-1 concentrations in studies with histological confirmation of MASLD are conflicting; in some studies, IGF-1 concentrations were lower in patients with more severe disease, including steatosis [58, 60], lobular inflammation or hepatocellular ballooning [55, 58, 69] and fibrosis [53–55, 69]. However, similar IGF-1 concentrations were observed regardless of the histological severity of hepatic steatosis [55, 65, 69], inflammation [53, 54, 60, 61, 65] or fibrosis [61, 65] in other studies (Table 2). A meta-analysis of 12 observational studies reported lower IGF-1 concentrations in patients with MASLD than in those without MASLD, as well as in patients with MASH than in those without MASH [75]. Interestingly, hepatic mRNA expression of IGF-1, but not of GHR, was lower in patients with MASH than in those with hepatic steatosis in one study [66]. Since directionality cannot be established in a cross-sectional study, these results may imply that lower IGF-1 pathway activation is associated with MASH, or that MASH impairs hepatic IGF-1 production. Lower IGF-1 mRNA expression, but not GHR or IGF-1 receptor mRNA expression, with increasing MASLD severity was also reported in a subsequent study [64]. In line with this, lower hepatic IGF-1 mRNA expression was observed with increasing severity of hepatic steatosis and inflammation in patients with long-standing human immunodeficiency virus (HIV) infection [76], a condition characterized by a high prevalence of steatotic liver disease [77]. It is also noteworthy that hepatic steatosis was associated with low IGF-1 and low adiponectin concentrations in a cross-sectional study of obese women without T2DM; in this study, low IGF-1 concentrations were independently associated with both hepatic steatosis and circulating adiponectin [78]. Although this observational study cannot show causality, as adiponectin seems to be associated with MASLD [79], the effect of the combination of low IGF-1 and low adiponectin on MASLD warrants further research.

Data on circulating GH concentrations were reported in only a subset of published studies (Table 2); this may be due to the high variability of circulating GH concentrations, being affected by multiple factors, including its physiologic pattern of pulsatile secretion, age, sex, body weight and adiposity, nutrition, and many diseases and medications [80], which render the interpretation of GH challenging. However, most studies showed lower circulating GH concentrations in patients with MASLD than in controls [14, 56, 57, 62], whereas one study reported similar GH concentrations in patients with MASLD and controls [60]. Notably, lower GH concentrations were observed in patients with MASH compared with those with non-MASH (i.e., hepatic steatosis and non-MASLD) [61], as well as in those with severe hepatic steatosis compared with those with mild or moderate hepatic steatosis [53]. 

There are also limited data on circulating concentrations of IGF-binding proteins (IGF-BPs) in patients with MASLD; IGF-BPs are ligands of IGF-1 in the circulation, thereby limiting IGF-1 binding and activating the IGF-1 receptor; IGF-BP3 is regarded as the main carrier of IGF-1 [65]. We previously showed that, although circulating total IGF-BP3 and IGF-BP4 were similar between histologically confirmed patients with hepatic steatosis and MASH, circulating intact IGF-BP3 and IGF-BP4 concentrations were higher in patients with MASH than in those with isolated hepatic steatosis [65]. Other investigators reported similar total IGF-BP3 concentrations in patients with and without MASLD [67], or higher IGF-BP3 concentrations in patients with than in those without MASLD [57]. More importantly, the ratio IGF-1/intact IGF-BP3 was lower in patients with MASH than in those with hepatic steatosis and remained robustly associated with MASH even after adjustment for IR, age and sex, whereas this association lost significance when BMI was added to the regression model [65]; this implies that BMI can be an important mediator of the association between IGF-1/intact IGF-BP3 ratio and MASH. Furthermore, this ratio was lower in the presence of hepatic fibrosis, but not in the presence of steatosis, lobular inflammation and hepatocellular ballooning [65]. Other investigators reported that IGF-BP5 concentrations were higher in patients with MASLD than in those without MASLD, as well as in those with definite MASH than in those with hepatic steatosis or possible MASH, and in patients with significant fibrosis than in those with early or no fibrosis; however, the IGF-BP5/IGF-1 ratio was similar across these groups [54]. Moreover, another study reported lower circulating IGF-BP1 concentrations in patients with MASLD than in those without MASLD [67]. In a *post-hoc* analysis of a randomized controlled trial (RCT) involving patients infected with HIV, hepatic IGF-BP6 and IGF-BP7 mRNA expression, as well as circulating IGF-BP7 concentrations, were positively associated with hepatic steatosis, inflammation and fibrosis [76]; however, this study lacked a control group, i.e., patients with HIV without MASLD.

Currently, there are no data on the association between adult GHD and MASH-related cirrhosis or HCC. In a systematic review of patients with cirrhosis of various etiologies, the association between low circulating IGF-1 concentrations and more severe liver disease was reported as a consistent finding; the authors also suggested that low IGF-1 concentrations may be an unfavorable prognostic factor in patients with cirrhosis [81]. Furthermore, in a prospective cohort study, circulating IGF-1 concentrations were lower in patients with hepatitis C virus-related cirrhosis who developed HCC [82]; the authors of this study supported that low IGF-1 concentrations may precede the diagnosis of HCC; therefore, it may be an early diagnostic marker [82], which, however, remains to be validated. Of course, these findings warrant the setting of relevant studies specifically in patients with MASLD-related cirrhosis and/or HCC.

Limitations of existing studies are their observational design, which cannot support causality, the use of hepatic ultrasonography in most of them, which may have led to misclassification bias, and, most importantly, the difference in BMI between groups in some of them, which, as mentioned above, is regarded as a major confounder of MASLD (Table 2).

Collectively, existing studies have shown lower or similar circulating GH and IGF-1 concentrations in patients with MASLD compared with those without MASLD. No study to date has shown higher circulating GH or IGF-1 concentrations in patients with MASLD than in those without MASLD. These results warrant cohort studies, ideally prospective ones, to elucidate the temporal associations of circulating GH, IGF-1 and IGF-1/IGF-BPs ratios, which better reflect bioavailable IGF-1, with the incidence and severity of MASLD. It seems that IGF-1 and/or IGF-BPs may contribute minimally to hepatic steatosis via their direct effects on hepatocytes, but they may prove useful biomarkers for MASLD, since circulating IGF-1 and IGF-BPs concentrations are affected by GHD.

## Recombinant GH and Relevant Treatment

Currently, limited data exist on the effect of rhGH treatment in patients with MASLD [50, 51, 83–89] (Table 3). The first case report (2007) described an adult with GHD and MASH who experienced histological improvement in hepatic steatosis, inflammation and fibrosis, together with normalization of liver function tests (LFTs) and improvement in lipid profile after 6 months of rhGH treatment [90]. A subsequent case series published in 2012 showed that a 6-month administration of rhGH in adults with GHD and MASLD reduced LFTs [51]. More importantly, in a subset of adults with MASH and paired liver biopsies, rhGH treatment improved hepatic steatosis and fibrosis, but not inflammation [51] (Table 3). Two years later, these results were validated in a retrospective cohort study of adults with GHD, showing that long-term (≥ 24 months) rhGH administration decreased LFTs and hyaluronic acid, a biomarker of hepatic fibrosis [86]. Subsequently, two single-center RCTs in overweight/obese patients with MASLD and low IGF-1 concentrations reported an improvement in hepatic steatosis after rhGH treatment [85, 87] (Table 3). Similar results were reported in another, non-randomized trial of patients with GHD [89]. It is important to note that a crossover study, in which healthy volunteers received rhGH and pegvisomant, a GH receptor antagonist, showed that GH acts, at least in part, by stimulating VLDL secretion from the liver, thereby reducing hepatic triglyceride content [84]. Contrary to the aforementioned studies, one retrospective study found that hepatic steatosis was not improved after rhGH treatment (*n* = 9) vs. no treatment (*n* = 9) [50]. Similarly, another study showed no effect of rhGH on hepatic steatosis (*n* = 12) [83]; however, the small sample size of these two studies should be considered when interpreting their negative results.

**Table 3:** Effect of RhGH or GHRH treatment on MASLD: evidence from interventional studies.*

First author Year Origin [Reference]†	Study design	Patients (*n*)	Medication; treatment duration	Main findings
Arlien-Søborg 2023 Denmark [83]	Single center, open-label, longitudinal case series; hepatic steatosis was evaluated with MRS	12 patients with GHD	rhGH subcutaneously; 21 (18–28) weeks	Hepatic steatosis was not reduced after treatment with rhGH.
Baumgartner 2025 Austria [84]	Single center, cross-over; hepatic steatosis was evaluated with MRS	10 healthy male volunteers	rhGH subcutaneously; 1week followed by pegvisomant subcutaneously; 1week; or vice versa	rhGH stimulated the secretion of VLDL from the liver. Hepatic *de novo* lipogenesis did not significantly change, although it was doubled after treatment with pegvisomant.
Dichtel 2023 USA [85]	Single center, double-blind, placebo controlled RCT; hepatic steatosis was evaluated with MRS and MRI-PDFF	53 NAFLD patients with overweight/obesity and low IGF-1 (27 on active arm; 26 on placebo arm)	rhGH subcutaneously; 6 months	Hepatic steatosis was reduced after treatment with rhGH (−5.2 ± 10.5%), but not after treatment with placebo (3.8 ± 6.9%). rhGH reduced ALT.
Matsumoto 2014 Japan [86]	Single center, open-label, retrospective cohort; NAFLD was evaluated with LFTs	50 patients with GHD (31 on active arm; 19 on no treatment arm)	rhGH subcutaneously; ≥24 months	rhGH reduced LFTs and circulating hyaluronic acid.
Meienberg 2016, UK [50]	Single center, open-label, retrospective cohort; NAFLD was evaluated with MRS	18 patients with GHD (9 on active arm; 9 on no treatment arm)	rhGH subcutaneously; 6 months	Hepatic steatosis was not statistically reduced more in the rhGH (−0.6%) than control group (+ 0.1%).
Nishizawa 2012 Japan [51]	Single center, open-label, longitudinal case series; hepatic steatosis was evaluated with US; liver biopsy was performed in selected patients only	11 patients with GHD and NAFLD; (5 with NASH)	rhGH subcutaneously; 6–12 months	Hepatic steatosis and fibrosis, albeit not lobular inflammation and hepatocellular ballooning, were improved in patients with NASH (*n* = 5). rhGH reduced LFTs.
Pan 2021 USA [87]	Single center, open-label RCT; hepatic steatosis was evaluated with MRS	24 NAFLD patients with obesity and low IGF-1 (13 on active arm; 11 on no treatment arm)	rhGH subcutaneously; 6 months	Hepatic steatosis was reduced after treatment with rhGH (−3.3%). Resolution of hepatic steatosis was observed in 56% vs. 11% in active and control arm, respectively. rhGH did not affect LFTs.
Stanley 2019 USA [88]	Multicenter, double-blind, placebo controlled RCT; hepatic steatosis was evaluated with MRS; hepatic fibrosis and NAS were evaluated histologically	61 NAFLD patients with HIV (31 on active arm; 30 on placebo)	Tesamorelin subcutaneously; 12 months	Hepatic steatosis was reduced after treatment with tesamorelin (−4.1%), but not after treatment with placebo (3.8 ± 6.9%). Resolution of hepatic steatosis was observed in 35% vs. 4% in active and placebo arm, respectively. Tesamorelin did not improve hepatic fibrosis or NAS more than placebo; however, hepatic fibrosis was progressed in less individuals in the active (11%) than the placebo (38%) arm. Tesamorelin did not affect LFTs.
Taguchi 2025 Japan [89]	Single center, open-label, non-randomized trial; hepatic steatosis was evaluated with MRI-PDFF	30 patients with GHD (20 on active arm; 10 on no treatment arm)	rhGH subcutaneously; 10.8 months (mean)	Hepatic steatosis was reduced after treatment with rhGH. rhGH reduced LFTs.

*: Original nomenclature of the liver disease (i.e., NAFLD, MAFLD, MASLD) was kept in the table for each study, for the sake of consistency

†: Studies are sorted in alphabetical order, primarily according to the first author surname and secondarily according to the publication year

Abbreviations: *ALT* alanine aminotransferase, *GHD* growth hormone deficiency, *GHRH* growth hormone releasing hormone, *HIV* human immunodeficiency virus, *IGF-1* insulin-like growth factor-1, *LFTs* liver function tests, *MAFLD* metabolic dysfunction-associated fatty liver disease, *MASLD* metabolic dysfunction-associated steatotic liver disease, *MRI-PDFF* magnetic resonance imaging-proton density fat fraction, *MRS* magnetic resonance spectroscopy, *n* number, *NAFLD* nonalcoholic fatty liver disease, *NAS* NAFLD activity score, *NASH* nonalcoholic steatohepatitis, *RCT* randomized controlled trial, *rhGH* recombinant human growth hormone, *US* ultrasonography, *VLDL *very low-density lipoprotein

Regarding tesamorelin, a GHRH analog, it was shown to improve hepatic steatosis and reduce visceral fat in a multicenter RCT involving patients with HIV and MASLD [88] (Table 3). Hepatic steatosis was also resolved at greater rates after treatment with rhGH [87] or tesamorelin [88] compared with no treatment [87] or placebo [88], respectively. Hepatic fibrosis and NAFLD activity score (NAS), histologically evaluated as secondary outcomes after tesamorelin treatment, were not improved when compared with placebo; however, tesamorelin was shown to prevent hepatic fibrosis progression at a higher rate than placebo (Table 3) [88]. A *post-hoc* analysis of this study [88] also showed that tesamorelin treatment increased circulating concentrations of IGF-BP1 and IGF-BP3, decreased IGF-BP2 and IGF-BP6, and did not affect IGF-BP7 [76]. Although these findings highlight that tesamorelin may affect the GH/IGF-1 axis at multiple levels, including circulating IGF-1 carriers, the clinical implications of this pharmacological effect, if any, remain to be elucidated.

In line with these results, two meta-analyses comprising a limited number of studies (*n* = 3) showed that GH augmentation reduced hepatic steatosis and visceral adipose tissue, whereas BMI, circulating glucose and lipid concentrations were not affected [91, 92]. These meta-analyses also showed that GH augmentation decreased alanine aminotransferase (ALT) [91, 92] and gamma-glutamyl transferase (GGT) [92], although existing studies provided conflicting results regarding LFTs (Table 3).

Regarding safety, treatment with rhGH [85, 87] or tesamorelin [88] were deemed safe in the relevant studies. More specifically, lower extremity edema [85] and headache [87] were more common in the rhGH-treated group than in the control group. Local reactions (pain, erythema, bruising or allergy) were occasionally reported at the injection sites, usually resolving spontaneously [85, 87]. Notably, fasting glucose [85, 87], fasting insulin and IR [85] remained unchanged after rhGH treatment. Regarding tesamorelin, two patients discontinued treatment due to hyperglycemia; local reactions at the site of injections were also observed [88]. Importantly, safety data from a registry-based study, the Hypopituitary Control and Complications Study (HypoCCS), showed that patients with adult-onset craniopharyngioma or pituitary adenoma and GHD (*n* = 4564) who received rhGH for ≥ 6 months showed lower (19%), albeit not statistically significant, rates of the composite hepatic outcome (that included but was not limited to MASLD) compared with patients not treated with rhGH (27%) [93]. However, it should be underlined that GHD was not isolated in the HypoCCS. In most cases, craniopharyngiomas or pituitary adenomas may also have led to hypothyroidism [94] or hypogonadism [95], which are associated with MASLD too. Furthermore, craniopharyngiomas or large pituitary adenomas may sometimes affect hypothalamus resulting in hypothalamic syndrome, characterized by uncontrollable appetite and obesity, as well as circadian rhythm disruption and autonomic dysfunction [96]. Therefore, in cases of not isolated GHD, MASLD may be multifactorial and should be considered as such.

It is also noteworthy that circulating concentrations and activity of fibroblast activation protein-α (FAPα) increase after treatment (3–6 months) with rhGH in patients with GHD [97]. Inversely, FAPα was shown to be elevated in patients with acromegaly and to decrease after the control of the disease [98]. Since FAPα is increased during inflammation, as well as in hepatic fibrosis and cirrhosis [97], further studies are needed to show whether FAPα increase after rhGH treatment may adversely affect hepatic fibrosis in patients with GHD, or whether this increase is associated with collagen breakdown.

Collectively, limited data show favorable effects of treatment with rhGH or tesamorelin on hepatic steatosis and possibly on hepatic fibrosis progression in adult patients with MASLD and GHD or those with low circulating IGF-1 concentrations (Table 3). However, further large studies are needed to draw more definite results. Until clearer answers are provided, the presence of MASLD in patients with GHD should not discourage rhGH replacement, as favorable effects on MASLD may occur. Of course, administration of rhGH or GHRH analogs in patients with MASLD and normal or high-normal IGF-1 concentrations is not recommended, as it might result in supraphysiologic IGF-1 concentrations with possible adverse clinical consequences.

## Closing Remarks

This review summarizes the currently available data on the link between adult GHD and MASLD. Concerning the pathophysiology, experimental data support a direct effect of GH on hepatocytes, predominantly through the JAK2-STAT5 pathway, a downstream of GHR [26], which reduces hepatic steatosis [26]. Therefore, GHD may contribute to hepatic steatosis, both directly and indirectly, the latter through the favorable effects of GH on systemic IR and MetS features, i.e., abdominal obesity, atherogenic dysglycemia, dyslipidemia and hypertension [12, 13, 18]. However, experimental data linking GHD with hepatic inflammation and fibrosis are scarce. It seems that IGF-1, by acting on HSCs, may reduce hepatic fibrosis [38], although additional studies are needed to corroborate these findings. Concerning clinical associations, MASLD is a highly prevalent condition in adults with GHD (Table 1). Vice versa, lower or similar circulating GH and IGF-1 concentrations are observed in patients with MASLD compared with those without MASLD in observational studies (Table 2). Regarding treatment, favorable effects of rhGH or tesamorelin on hepatic steatosis and, possibly, on hepatic fibrosis progression in patients with MASLD and GHD, or those with MASLD and low circulating IGF-1 concentrations, are reported in interventional studies (Table 3).

Contrary to GHD, acromegaly is a rare endocrine disease characterized by GH excess, leading to elevated circulating IGF-1 concentrations [99]. Patients with acromegaly have low visceral and subcutaneous adipose tissue mass, whereas skeletal muscle mass is high; interestingly, intramuscular adipose tissue is increased in patients with acromegaly, possibly contributing to their severe IR [100, 101]. Despite IR, patients with active acromegaly have lower rates of MASLD than those with controlled acromegaly; notably, surgical treatment of acromegaly increases adipose tissue and hepatic steatosis [101, 102]. Notably, the addition of pegvisomant, which, as mentioned above, is a GH receptor antagonist, to a somatostatin analog worsened hepatic steatosis in patients with acromegaly, despite a reduction in the somatostatin analog dose [103].

Although current data do not support the development of relevant guidelines, the high prevalence of MASLD in patients with adult GHD (Table 1) may warrant screening for MASLD in this patient population. Treatment with rhGH is common in children and adolescents with GHD, but it may also be considered in selected patients with adult GHD, as these patients may benefit in terms of obesity and other MetS components, as well as exercise capacity, skeletal mass, and quality of life [104]. In this regard, adults with GHD and MASLD may also benefit in terms of hepatic disease (Table 3); however, studies specifically designed to investigate the long-term effects of rhGH treatment on the progression of MASLD to advanced disease and the risk of mortality (all-cause, cardiovascular-related, cancer-related and liver-related) would be important to clarify the possibly hepatoprotective effects of rhGH treatment in MASLD. Of course, the administration of rhGH in patients with MASLD without GHD is contraindicated, since it may result in adverse events, including drug-induced acromegaly.

In this review, we did not examine studies on the association between pediatric GHD and MASLD because the data are, to date, limited and contradictory. As GHD seriously affects the stature and weight in children (low weight), this may mask any adverse effects of GHD on MASLD, thus rendering existing observational data on the association between GHD and MASLD in children difficult to interpret.

In conclusion, MASLD is highly prevalent in patients with adult GHD and treatment with rhGH may exert favorable effects on hepatic steatosis and possibly on hepatic fibrosis progression in patients with MASLD and GHD. However, more specifically designed studies are needed to carefully assess the risk–benefit ratio of rhGH replacement in adults with GHD and MASLD. In this regard, we believe that a multidisciplinary approach, ensuring close collaboration between endocrinologists and hepatologists, is clinically important for the management of patients with adult GHD and MASLD.

## Key References

- Kong T, Gu Y, Sun L, Zhou R, Li J, Shi J. Association of nonalcoholic fatty liver disease and growth hormone deficiency: a systematic review and meta-analysis. Endocr J. 2023;70:959 − 67. 10.1507/endocrj.EJ23-0157.
  - ○ A meta-analysis of 10 observational studies highlighting the prevalence of MASLD in patients with GHD.
- Dutta D, Nagendra L, Mohindra R, Bhattacharya S, Joshi A, Kamrul-Hasan A. Role of Growth Hormone Therapy in Metabolic-Dysfunction-Associated Steatotic Liver Disease: A Systematic Review and Meta-Analysis. Indian J Endocrinol Metab. 2024;28:336 − 42. 10.4103/ijem.ijem_488_23.
  - ○ A meta-analysis of three RCTs showing that treatment with rhGH reduced intrahepatic lipid content and visceral adiposity.
- Mohamed I, Gautam M, Abosheaishaa H, Hussain S, Kumar K, Kotak A, et al. Growth hormone augmentation in metabolic dysfunction-associated steatotic liver disease: a systematic review and meta-analysis of randomized controlled trials. Eur J Gastroenterol Hepatol. 2024;36:1259-66. 10.1097/meg.0000000000002819.
  - ○ A meta-analysis of three RCTs showing that rhGH treatment reduced hepatic fat fraction.
- Vázquez-Borrego MC, Del Río-Moreno M, Pyatkov M, Sarmento-Cabral A, Mahmood M, Pelke N, et al. Direct and systemic actions of growth hormone receptor (GHR)-signaling on hepatic glycolysis, de novo lipogenesis and insulin sensitivity, associated with steatosis. Metabolism. 2023;144:155589. 10.1016/j.metabol.2023.155589.
  - ○ An experimental study highlighting the direct and indirect metabolic effects of growth hormone receptor signaling on the liver.
- Fellinger P, Beiglböck H, Semmler G, Pfleger L, Smajis S, Baumgartner C, et al. Increased GH/IGF-I Axis Activity Relates to Lower Hepatic Lipids and Phosphorus Metabolism. J Clin Endocrinol Metab. 2023;108:e989-e97. 10.1210/clinem/dgad206.
  - ○ After evaluation of the GH/IGF-1 axis in a fasting state and during an oral glucose tolerance test in metabolically healthy individuals, it was shown that lower GH/IGF-1 axis activity was associated with higher intrahepatic lipid content.
- Taguchi T, Ito S, Fujishima R, Shimizu N, Hagiwara W, Matoba K, et al. Hypertriglyceridemia and younger age are associated with effectiveness of growth hormone therapy on hepatic steatosis. Endocr J. 2025;72:355 − 64. 10.1507/endocrj.EJ24-0481.
  - ○ Treatment with rhGH in adults with GHD reduced intrahepatic lipid content and improved serum lipid profile.

## Data Availability

No datasets were generated or analysed during the current study.

## Declarations

### Originality of figures and tables

All figures and tables are original and were developed specifically for this article; therefore, no permission to reproduce is required.
